# Short-term Patient-Reported Outcomes After Primary Anterior Cruciate Ligament Reconstruction With and Without Lateral Extra-articular Tenodesis: A Matched-Cohort Analysis From the Swedish Knee Ligament Registry

**DOI:** 10.1177/03635465261456637

**Published:** 2026-06-25

**Authors:** James A. Pruneski, Bálint Zsidai, Felix Öttl, Kristian Heder Ternell, Riccardo Cristiani, Volker Musahl, Eric Hamrin Senorski, Kristian Samuelsson, Alexandra Horvath

**Affiliations:** * Department of Orthopaedic Surgery, Tripler Army Medical Center, Honolulu, Hawaii, USA; † Sahlgrenska Sports Medicine Center, Gothenburg, Sweden; ‡ Department of Orthopaedics, Institute of Clinical Sciences, Sahlgrenska Academy, University of Gothenburg, Gothenburg, Sweden; § Department of Orthopaedics, Skåne University Hospital, Malmö/Lund, Sweden; ‖ Department of Orthopaedic Surgery, Balgrist University Hospital, University of Zürich, Zurich, Switzerland; ¶ Department of Orthopaedics, Sahlgrenska University Hospital, Mölndal, Sweden; # Department of Molecular Medicine and Surgery, Section of Sports Medicine, Karolinska Institutet, Stockholm, Sweden; ** Stockholm Sports Trauma Research Center (SSTRC), FIFA Medical Centre of Excellence, Stockholm, Sweden; †† Department of Orthopaedic Surgery, UPMC Freddie Fu Sports Medicine Center, Pittsburgh, Pennsylvania, USA; ‡‡ Unit of Physiotherapy, Department of Health and Rehabilitation, Institute of Neuroscience and Physiology, Sahlgrenska Academy, University of Gothenburg, Gothenburg, Sweden; §§ Sportrehab Sports Medicine Clinic, Gothenburg, Sweden; Investigation performed at Sahlgrenska Sports Medicine Center, Gothenburg, Sweden

**Keywords:** anterior cruciate ligament reconstruction, lateral extra-articular tenodesis, patient-reported outcomes, KOOS, Swedish Knee Ligament Registry

## Abstract

**Background:**

Despite reduced graft failure rates reported in randomized controlled trials comparing anterior cruciate ligament reconstruction (ACL-R) with and without lateral extra-articular tenodesis (LET), the impact of LET on postoperative patient-reported outcomes (PROs) remains poorly defined.

**Purpose:**

To compare short-term PROs, as well as anterior cruciate ligament (ACL) revision rates and clinical failure rates (defined as Knee injury and Osteoarthritis Outcome Score [KOOS] Quality of Life [QoL] value <44) between patients undergoing primary ACL-R with and without concomitant LET.

**Study Design:**

Cohort study; Level of evidence, 3.

**Methods:**

Data were extracted from the Swedish Knee Ligament Registry for all patients undergoing primary ACL-R between January 1, 2005, and June 25, 2025. Patients who underwent ACL-R + LET were matched to patients who underwent ACL-R without LET using 1:4 matching based on age, sex, body mass index, graft type, time from injury to reconstruction, and concomitant meniscal and cartilage injury status. The primary outcomes were KOOS values at the 1- and 2-year follow-ups.

Secondary outcomes included revision ACL-R and clinical failure within 1 and 2 years. Categorical variables were compared using Fisher exact or chi-square tests, continuous variables were compared using Fisher nonparametric permutation tests, and within-group KOOS changes were compared using Wilcoxon signed-rank tests. Adjusted 1- and 2-year KOOS Symptoms comparisons for baseline differences and outcomes were interpreted relative to published minimal clinically important difference thresholds.

**Results:**

After matching, the final cohort consisted of 870 patients: 174 in the ACL-R + LET group and 696 in the ACL-R group. There were no significant between-group differences in KOOS_4_ (mean of the Symptoms, Pain, Sports and Recreation, and QoL subscales) scores at the 1-year (77.7 ± 15.2 for ACL-R + LET vs 78.8 ± 14.9 for ACL-R) or 2-year (79.9 ± 16.7 for ACL-R + LET vs 80.7 ± 13.4 for ACL-) follow-up (*P* > .5). Both groups demonstrated significant within-group improvements in KOOS_4_ scores from baseline through the 2-year follow-up (all *P*≤ .002). ACL revision rates at 2 years were 2.3% and 2.4% in the ACL-R + LET and ACL-R groups, respectively. Rates of clinical failure at 2 years were similar between groups (25.5% for ACL-R + LET vs 25.6% for ACL-R; *P* > .99).

**Conclusion:**

In this matched-cohort registry study, the addition of LET to primary ACL-R did not demonstrate superior short-term PROs at 1 or 2 years postsurgery.

The primary goals of anterior cruciate ligament reconstruction (ACL-R) are to restore knee stability, reduce the risk of secondary intra-articular injury, and enable patients to return to premorbid levels of sport and physical activity. Despite the widespread success of modern ACL-R techniques, a clinically meaningful proportion of patients continues to experience residual anterolateral rotatory laxity and instability after reconstruction. In fact, up to 25% of patients demonstrate a persistent positive pivot-shift test on clinical examination after ACL-R, and approximately 6% sustain secondary ipsilateral graft rupture within 2 years of the index procedure.^9,15,16,21^ These persistent complications have driven ongoing investigation into supplementary surgical strategies that can augment rotatory control of the anterior cruciate ligament (ACL)–reconstructed knee.

Lateral extra-articular tenodesis (LET) was first described by Lemaire^17^ in 1967 as a stand-alone surgical treatment for anterolateral knee instability, and it has experienced a marked resurgence over the past decade commensurate with a renewed anatomic interest in the anterolateral complex of the knee.^20^ When performed concomitantly with ACL-R, LET seems to reduce anterolateral rotatory laxity and mechanical demands on the intra-articular graft, potentially improving graft integration and long-term survival.^20,26^ The landmark STABILITY randomized controlled trial (RCT) demonstrated that the addition of a modified Lemaire LET to single-bundle hamstring tendon ACL-R in young patients at high risk for failure resulted in a clinically meaningful reduction in ACL-R clinical failure and graft rupture rates at 2 years.^9^ Subsequent systematic reviews and meta-analyses of RCTs have corroborated these findings, demonstrating that the addition of a lateral extra-articular procedure (LEAP) to primary ACL-R improves residual anterolateral rotatory laxity and reduces graft rerupture rates relative to ACL-R alone.^14,20^ In elite athletes, a recent meta-analysis reported a >60% reduction in ACL rerupture risk when an LEAP was added to ACL-R, further supporting its protective role in high-demand populations.^6^

Despite this growing evidence base, the question of which patients should undergo LET as an adjunct to primary ACL-R remains actively debated. Importantly, high-level evidence regarding the utility of LET in nonhamstring grafts is limited.^2,3,25^ While the benefits of LET in specifically selected high-risk patients are increasingly accepted, concerns persist regarding the potential for short-term morbidity and the theoretical risk of overconstraint and accelerating degenerative change in the lateral compartment over the long term.^2,30^ Critically, evidence examining the effect of LET on patient-reported outcomes (PROs) has been less consistent than evidence supporting its effect on graft failure and rotatory laxity, as a recent systematic review and meta-analysis of RCTs found no significant between-group difference in patient-reported function at 1 or 2 years after surgery, despite reduced laxity in the LET cohort.^14^ Taken together, these findings suggest that the indications for LET remain incompletely defined, and its application in routine clinical practice may extend beyond the patient populations for whom the strongest evidence exists.^32^

Large-scale national registry studies offer a unique opportunity to examine outcomes of LET in real-world, unselected patient populations not constrained by strict trial eligibility criteria. The Swedish Knee Ligament Registry (SKLR) is a prospective, nationwide clinical database that collects data on patients undergoing ACL-R, representing one of the most comprehensive repositories of ACL-related surgical outcomes and PROs globally.^1,11^ The purpose of the current study was to compare short-term PROs and revision ACL-R rates between patients undergoing primary ACL-R with and without concomitant LET in a matched cohort derived from the SKLR at 1 and 2 years postoperatively. We hypothesized that the addition of an LET to ACL-R would not improve short-term PROs across a broad national cohort.

## Methods

### Study Design, Data Source, and Ethical Aspects

This study was based on data from the SKLR, a prospective nationwide clinical database established in 2005 to collect data on patients undergoing reconstructive knee ligament surgery in Sweden.^1,11^ Data collection involves a 2-part process: patients contribute preoperative and postoperative knee function data through mail- or web-based surveys at intervals of 1, 2, 5, and 10 years after surgery, while surgeons report surgical intervention, technique, and concomitant injury details. Ethical approval was granted by the Swedish ethical review authority (Dnr 2022-00913-01), and the study was performed in accordance with the Declaration of Helsinki and reported in accordance with the STROBE (Strengthening the Reporting of Observational Studies in Epidemiology) checklist.^31^

### Patient Selection and Eligibility Criteria

Patients registered for primary ACL-R in the SKLR between January 1, 2005, and June 25, 2025, were assessed for eligibility. Inclusion criteria were (1) primary ACL-R with or without LET, (2) age 14 to 60 years at the time of surgery, and (3) complete preoperative Knee injury and Osteoarthritis Outcome (KOOS) data. Patients were excluded for (1) concomitant ligamentous injuries (posterior cruciate ligament, medial or lateral collateral ligament, posterolateral corner), (2) concomitant fractures (other than Segond fractures or subchondral impaction fractures), (3) quadriceps or patellar tendon injuries, (4) revision ACL-R at index surgery in SKLR, or (5) missing time from injury to-revision data. A minimum 2-year elapsed follow-up time from reconstruction was required for inclusion; this criterion was applied to follow-up time (calendar duration from reconstruction), not to questionnaire return. Accordingly, patients eligible for the 2-year KOOS questionnaire who had not yet returned their questionnaire at the time of data extraction were retained in the matched cohort for all analyses and contributed data only for time points at which questionnaires were returned.

### Matching Procedure

To minimize confounding and ensure comparability between groups, patients with ACL-R + LET were matched to patients with ACL-R without LET using caliper matching with a ratio of 1:4. Matching variables included age at primary surgery, sex, body mass index (BMI), graft type (hamstring tendon vs nonhamstring tendon), time from injury to reconstruction, medial and lateral meniscus injury status, and cartilage injury status.

### Study Variables

Baseline descriptive variables included age, sex, BMI, and smoking status. Injury-related variables included injury setting (categorized as alpine/skiing, pivoting sport, nonpivoting sport, other physical activity, traffic related, and other) and time from injury to surgery. Surgical variables included graft type, concomitant meniscal injury status and treatment (resection or repair), and concomitant cartilage injury and LET technique (eg, modified Lemaire vs MacIntosh/Arnold-Coker variant). Inclusion of the LET technique as a variable in the SKLR began in 2016 due to increasing adoption. Patients who underwent revision ACL-R at any hospital or clinic in Sweden during the study period were identified through the SKLR using their unique Swedish personal identity number.^18^

### Outcome Measures

The primary outcome was subjective knee function assessed using the KOOS at 1 and 2 years of follow-up. The KOOS consists of 5 subscales: Symptoms, Pain, Activities of Daily Living (ADL), Sports and Recreation (Sports/Rec), and Quality of Life (QoL), each scored 0 to 100, with 100 indicating no symptoms).^24^ A composite score, the KOOS_4_ (the mean of the Symptoms, Pain, Sports/Rec, and QoL subscales excluding ADL to avoid a ceiling effect in the predominantly young, active ACL-R population), was used as the primary summary metric, as originally described and validated by Frobell et al.^7^

The secondary outcomes were (1) ipsilateral ACL revision, defined as revision ACL-R on the operated knee within 1 and 2 years; and (2) clinical failure, defined as a KOOS QoL subscale score <44. This cutoff value has previously been described as associated with more than moderately decreased knee-related quality of life and has been shown to be associated with an increased risk of revision ACL-R.^7,10^

### Statistical Analysis

After deidentification, statistical analysis was performed using SAS/STAT Version 9.4 (SAS Institute) by a consultant statistician with formal training. Categorical variables are reported as frequencies with percentages, and continuous variables are reported as mean ± standard deviation, and mean (95% confidence interval). Between-group comparisons were performed using the Fisher exact test for dichotomous variables and chi-square test for nonordered categorical variables. The Fisher nonparametric permutation test was used for continuous variables because of its flexibility in handling the heterogeneous distributions and small group sizes encountered in registry data, where asymptotic parametric assumptions are unreliable due to bounding and ceiling effects. Within-group comparisons of KOOS values over time were performed using the Wilcoxon signed-rank test. Effect sizes were calculated using Cohen *d* (N ≥ 50) or Hedges *g* (N < 50); values were interpreted as <0.2 (trivial), 0.2 to 0.5 (small), 0.5 to 0.8 (moderate), and >0.8 (large).

Between-group KOOS Symptoms values at the 1- and 2-year follow-ups were additionally analyzed using analysis of covariance (ANCOVA) with preoperative KOOS Symptoms value as a covariate, to address the statistically significant baseline imbalance in this subscale. The ANCOVA results are reported alongside unadjusted comparisons.

For clinical contextualization, between-group differences and within-group improvements were compared against published minimal important change (MIC; within group) and minimal clinically important difference (MCID; between groups) thresholds for KOOS Sports/Rec (12.1 points)^13^ and KOOS QoL (18.3 points)^13,19^ in the ACL-R group, as these values are suggested to be the most robust in the literature. Using the established MIC values derived from the Norwegian Knee Ligament Registry study for patients undergoing ACL-R, we estimated the KOOS_4_ MIC as 8 to 10 points.^13^

As this study used existing prospectively collected registry data, the available sample was constrained by the registry rather than by recruitment. An a priori power calculation was performed to confirm that the matched cohort was adequately sized to detect a clinically meaningful between-group difference in the primary outcome (KOOS_4_ at 2 years). On the basis of previously published ACL-R cohorts,^7,9^ a between-group standard deviation for KOOS_4_ of 17 points and an MIC of 10 points were assumed.^13^ Using a 2-sided α value of .05, target power of 0.80, and the planned 1:4 caliper-matching ratio (ACL-R + LET to ACL-R), we calculated the minimum sample size required to detect a 10-point between-group difference to be 29 patients in the ACL-R + LET arm and 114 patients in the ACL-R arm (total 143).

## Results

### Patient Characteristics

**Figure 1.**
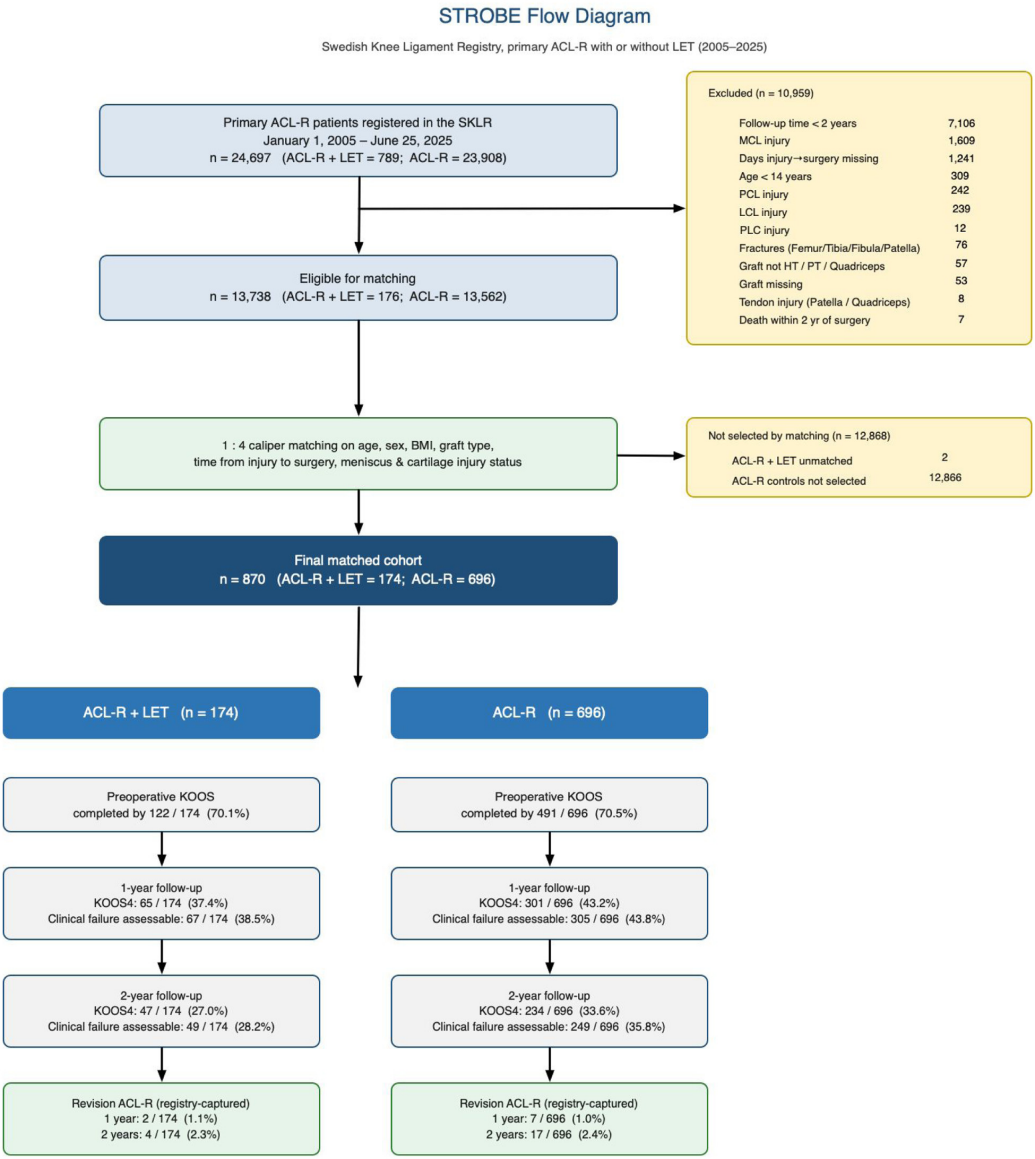
STROBE (Strengthening the Reporting of Observational Studies in Epidemiology) flow diagram. Revision anterior cruciate ligament reconstruction (ACL-R) events are captured at the population level via the Swedish personal identity number and are therefore available for all 870 matched patients regardless of Knee injury and Osteoarthritis Outcome Score (KOOS) questionnaire return. BMI, body mass index; HT, hamstring tendon; KOOS_4_, mean of the Symptoms, Pain, Sports and Recreation, and Quality of Life subscales; LCL, lateral collateral ligament; LET, lateral extra-articular tenodesis; MCL, medial collateral ligament; PCL, posterior cruciate ligament; PLC, posterolateral corner; PT, patellar tendon; SKLR, Swedish Knee Ligament Registry.

A total of 24,697 patients were registered in the SKLR during the study period. After applying the eligibility criteria, 13,738 patients (176 with LET and 13,562 without LET) were eligible for matching (Figure 1). After the 1:4 matching procedure, the final cohort consisted of 870 patients: 174 in the ACL-R + LET group and 696 in the ACL-R group (Table 1). The mean age at surgery was 25.1 ± 11 years for the ACL-R + LET group and 25.2 ± 9.5 years for the ACL-R group (*P* = .85). Both groups had similar sex distribution (56% male; *P* > .99) and mean BMI (24.8 ± 4.1 kg/m^2^ for ACL-R + LET and 24.7 ± 3.7 kg/m^2^ for ACL-R; *P* = .82). The mean time from injury to surgery was 26.9 ± 46.0 months for the ACL-R + LET group and 21.3 ± 35.4 months for the ACL-R group (*P* = .12). Activity that led to ACL injury differed significantly between groups (overall *P* = .043), with pivoting sports as the predominant setting in both groups (67% of the ACL-R + LET group and 70% of the ACL-R group), but a notably higher proportion of injuries in other settings (eg, workplace injury, fall at home) in the ACL-R + LET group (14% vs 6.9%; *P* = .005). Graft type distribution was identical between groups (hamstring tendon: 67.2% for both groups; patellar tendon: 16.7% for the ACL-R + LET group, 16.8% the ACL-R group; quadriceps tendon, 16.1% for the ACL-R + LET group, 15.9% for the ACL-R group; *P* > .99). Concomitant meniscal and cartilage injury rates were balanced between groups (*P* > .99 for all).

**Table 1:** Baseline Patient Characteristics*^*a*^*

	ACL-R	ACL-R + LET	*P* Value*^*b*^*
	(n = 696)	(n = 174)	
Descriptive characteristics			
Age, y	25.2 ± 9.5	25.1 ± 11	.85
Male sex	388 (56)	97 (56)	>.99
Smoking	8 (1.1)	4 (2.3)	.26
BMI, kg/m^2^	24.7 ± 3.7	24.8 ± 4.1	.82
Injury			
Time from injury to surgery, mo	21.3 ± 35.4	26.9 ± 46.0	.12
Injury setting			
Alpine/skiing	99 (14.2)	17 (9.8)	.14
Pivoting sport	490 (70.4)	116 (66.7)	.36
Nonpivoting sport	28 (4.0)	10 (5.7)	.30
Other physical activity	25 (3.6)	6 (3.4)	>.99
Traffic related	6 (0.9)	1 (0.6)	>.99
Other	48 (6.9)	24 (14)	**.005**
Surgical			
Graft type			
Patellar tendon	117 (16.8)	29 (16.7)	>.99
Hamstring tendons	468 (67.2)	117 (67.2)	
Quadriceps tendon	111 (15.9)	28 (16.1)	
Concomitant meniscal injury	432 (62.1)	108 (62.1)	>.99
Concomitant cartilage injury	124 (17.8)	31 (17.8)	>.99
Outcomes			
Revision ACL surgery within 1 y	7 (1.0)	2 (1.1)	>.99
Revision ACL surgery within 2 y	17 (2.4)	4 (2.3)	>.99

a Data are reported as n (%) or mean ± SD. ACL, anterior cruciate ligament; ACL-R, anterior cruciate ligament reconstruction; BMI, body mass index; LET, lateral extra-articular tenodesis.

b *P* values were derived from the Fisher exact test (dichotomous variables), chi-square test (nonordered categorical variables), or Fisher nonparametric permutation test (continuous variables). Boldface type indicates statistical significance.

### Baseline Subjective Knee Function

Preoperative KOOS subscale values are presented in Table 2. At baseline, patients in the ACL-R + LET group reported significantly higher KOOS Symptoms values (74.4 ± 19.5 vs 70.1 ± 19.2; *P* = .016; *d* = 0.23). No significant between-group differences were observed for Pain, ADL, Sports/Rec, QoL, or KOOS_4_ (*P*≥ .25).

**Table 2:** KOOS Subscale Values Between Groups at Baseline and 1 and 2 Years of Follow-up*^*a*^*

	ACL-R	ACL-R + LET	*P* Value*^*b*^*	Difference, Mean (95% CI)	Effect Size*^*c*^*
	(n = 696)	(n = 174)			
Preoperative KOOS					
Symptoms*^*d*^*	70 ± 19.2	74 ± 19.5	**.016**	−4.4 (−8.36 to −0.50)	0.23
Pain	75 ± 17.9	77 ± 19.9	.35	−1.7 (−5.38 to 1.86)	**0.09**
ADL	84 ± 17.3	84 ± 17.8	.85	−0.34 (−3.90 to 3.17)	**0.02**
Sports/Rec	43 ± 28.9	45 ± 32.5	.61	−1.6 (−7.45 to 4.79)	**0.05**
QoL	37 ± 21.6	39 ± 28.7	.46	−1.9 (−6.52 to 2.85)	**0.08**
KOOS_4_	67 ± 16.0	69 ± 18.6	.25	−2.0 (−5.32 to 1.19)	**0.12**
1-y follow-up KOOS					
Symptoms	78 ± 17.2	75 ± 18.8	.21	3.2 (−1.89 to 7.84)	**0.18**
Pain	85 ± 15.0	86 ± 13.9	.67	−0.92 (−5.29 to 2.94)	**0.06**
ADL	93 ± 12.6	92 ± 16.6	.62	0.92 (−2.94 to 4.21)	**0.07**
Sports/Rec	67 ± 26.0	68 ± 24.0	.84	−0.69 (−7.96 to 6.44)	**0.03**
QoL	60 ± 22.8	58 ± 23.0	.62	1.6 (−4.75 to 7.84)	**0.07**
KOOS_4_	79 ± 14.9	78 ± 15.2	.59	1.2 (−3.17 to 5.16)	**0.08**
2-y follow-up KOOS					
Symptoms	79 ± 16.0	79 ± 17.7	.87	0.47 (−4.83 to 5.28)	**0.02**
Pain	87 ± 12.8	86 ± 17.3	.65	1.03 (−3.56 to 5.19)	**0.07**
ADL	94 ± 9.2	92 ± 15.0	.19	2.26 (−1.33 to 5.28)	**0.19**
Sports/Rec	70 ± 24.1	67 ± 25.4	.5	2.77 (−5.26 to 10.26)	**0.11**
QoL	63 ± 22.2	63 ± 23.3	.9	−0.45 (−7.77 to 6.84)	**0.02**
KOOS_4_	81 ± 13.4	80 ± 16.7	.74	0.80 (−3.89 to 5.14)	**0.06**

a Data are presented as mean ± SD unless otherwise indicated. Boldface type indicates statistical significance. ACL-R, anterior cruciate ligament reconstruction; ADL, Activities of Daily Living; KOOS, Knee injury and Osteoarthritis Outcome Score; KOOS_4_, mean of the Symptoms, Pain, Sports and Recreation, and Quality of Life subscales; LET, lateral extra-articular tenodesis; QoL, Quality of Life; Sports/Rec, Sports and Recreation.

b *P* values were derived from the Fisher nonparametric permutation test for continuous outcomes and the Fisher exact test for categorical outcomes. Differences in means and 95% CIs are based on the inversion of the Fisher nonparametric permutation test.

c Effect size: Cohen *d* (N ≥ 50) or Hedges *g* (N < 50), defined as the absolute difference in means divided by the pooled standard deviation. Interpretation: <0.20, trivial; 0.20 to 0.50, small; 0.50 to 0.80, moderate; >0.80, large.

d Statistically significant baseline difference in favor of ACL-R + LET (higher preoperative Symptoms value). Analysis of covariance -adjusted comparison at the 1-year follow-up: adjusted between-group difference −2.8 points (95% CI, −7.1 to 1.5; *P* = .20); at 2-year follow-up: −0.6 points (95% CI, −5.8 to 4.7; *P* = .83).

### KOOS Outcomes at Follow-up

#### Between-Group Comparisons

At the 1-year follow-up, there were no significant between-group differences for any KOOS subscale values (Table 2). All effect sizes were trivial (*d* < 0.20) to small (*d* < 0.50). All between-group KOOS_4_ differences were below the MCID of 8 points.^13^ The ANCOVA-adjusted analysis for KOOS Symptoms at 1 year, with preoperative Symptoms value as a covariate, yielded an adjusted between-group difference of −2.8 points (95% CI, −7.1 to 1.5; *P* = .20), confirming the absence of a significant between-group difference.

At the 2-year follow-up, no significant between-group differences were observed (Table 2). The observed between-group KOOS_4_ difference was 0.8 points, well below both the established MCID of 8 points and the minimum detectable difference of 6.3 points at 80% power (post hoc analysis).^13^ ANCOVA-adjusted analysis for Symptoms at 2 years yielded an adjusted between-group difference of −0.6 points (95% CI, −5.8 to 4.7; *P* = .83).

#### Within-Group Comparisons

In the ACL-R group, all KOOS subscale values improved significantly from baseline to the 1-year follow-up (all *P* < .001), with within-group improvements exceeding MIC thresholds for Sports/Rec and KOOS_4_ (Table 3). All subscale values improved significantly from baseline to the 2-year follow-up (all *P* < .001), with large effect sizes for Sports/Rec (1.07), QoL (1.44), and KOOS_4_ (1.03). No significant changes were observed from the 1-year to 2-year follow-up in the ACL-R group.

**Table 3:** Within-Group KOOS Subscale Changes Over Time: ACL-R Group (n = 696)*^*a*^*

	Change, Preoperative to 1-Y Follow-up			Change, Preoperative to 2-Y Follow-up		Change, 1-Y to 2-Y Follow-up	
	Mean (95% CI)	*P* Value	ES	Mean (95% CI)	*P* Value	Mean (95% CI)	*P* Value
KOOS Symptoms	9.73 (7.0 to 12.5)	**<.001**	0.51	10.3 (7.5 to 13.1)	**<.001**	0.19 (−2.18 to 2.54)	.76
KOOS Pain	11.0 (8.8 to 13.2)	**<.001**	0.64	11.9 (9.5 to 14.3)	**<.001**	−0.18 (−2.14 to 1.77)	.63
KOOS ADL	8.78 (6.75 to 10.8)	**<.001**	0.55	8.72 (6.49 to 11.0)	**<.001**	−0.76 (−2.23 to 0.68)	.30
KOOS Sports/Rec	24.9 (20.8 to 29.0)	**<.001**	0.89	28.7 (24.3 to 33.0)	**<.001**	0.30 (−3.07 to 3.58)	.39
KOOS QoL	25.1 (21.7 to 28.5)	**<.001**	1.23	27.4 (23.8 to 31.0)	**<.001**	1.92 (−1.26 to 5.06)	.079
KOOS_4_	13.7 (11.5 to 15.9)	**<.001**	0.90	14.6 (12.4 to 16.9)	**<.001**	0.31 (−1.57 to 2.21)	.17

a Preoperative mean (SD): Symptoms, 70.1 (19.2); Pain, 75.0 (17.9); ADL, 84.0 (17.3); Sports/Rec, 43.6 (28.9); QoL, 36.8 (21.6); KOOS_4_, 66.5 (16.0). Change data were available for patients with paired questionnaire responses at each interval. Within-group differences were assessed using the Wilcoxon signed-rank test. Effect sizes were calculated using Cohen *d* (absolute difference in means/pooled SD at preoperative time point). Interpretation: <0.20, trivial; 0.20 to 0.50, small; 0.50 to 0.80, moderate; >0.80, large. Boldface type indicates statistical significance. ACL-R, anterior cruciate ligament reconstruction; ADL, Activities of Daily Living; ES, effect size; KOOS, Knee injury and Osteoarthritis Outcome Score; KOOS_4_, mean of the Symptoms, Pain, Sports and Recreation, and Quality of Life subscales; QoL, Quality of Life; Sports/Rec, Sports and Recreation.

In the ACL-R + LET group, significant within-group improvements were observed from baseline to the 1-year follow-up for Pain, ADL, Sports/Rec, QoL, and KOOS_4_ (*P* < .001), with within-group improvements exceeding MIC thresholds for Sports/Rec and KOOS_4_. No change in Symptoms from baseline to 1 year was noted, possibly reflecting the higher baseline Symptoms value in this group (74.4 ± 19.5). Notably, the LET group demonstrated statistically significant further improvement in Symptoms (+5.7 points; *P* = .02), QoL (+7.5 points; *P* = .01), and KOOS_4_ (+4.2 points; *P* = .02) from 1 to 2 years, a pattern not observed in the ACL-R group (Table 4).

**Table 4:** Within-Group KOOS Subscale Changes Over Time: ACL-R + LET Group (n = 174)*^*a*^*

	Change, Preoperative to 1-Y Follow-up			Change, Preoperative to 2-Y Follow-up		Change, 1-Y to 2-Y Follow-up	
	Mean (95% CI)	*P* Value	ES	Mean (95% CI)	*P* Value	Mean (95% CI)	*P* Value
KOOS Symptoms	4.54 (−1.79 to 10.7)	.14	0.25	6.30 (−0.99 to 13.5)	.13	5.67 (1.26 to 10.1)	**.019**
KOOS Pain	11.3 (5.4 to 17.0)	**<.001**	0.55	9.14 (1.85 to 16.2)	**.012**	1.31 (−2.04 to 4.63)	.26
KOOS ADL	8.39 (2.17 to 14.6)	**<.001**	0.43	8.62 (1.75 to 15.44)	**.009**	2.29 (−2.52 to 7.94)	.95
KOOS Sports/Rec	21.9 (13.1 to 30.5)	**<.001**	0.71	25.9 (14.4 to 37.5)	**<.001**	2.94 (−5.38 to 11.4)	.54
KOOS QoL	21.9 (13.0 to 30.8)	**<.001**	0.78	21.6 (11.5 to 32.1)	**<.001**	7.54 (2.08 to 113.0)	**.011**
KOOS_4_	11.7 (6.3 to 16.9)	**<.001**	0.66	11.7 (4.7 to 18.8)	**.002**	4.20 (0.93 to 7.55)	**.016**

a Preoperative mean (SD): Symptoms, 74.4 (19.5); Pain, 76.7 (19.9); ADL, 84.3 (17.8); Sports/Rec, 45.2 (32.5); QoL, 38.6 (28.7); KOOS_4_, 68.5 (18.6). Change data were available for patients with paired questionnaire responses at each interval. Within-group differences were assessed using the Wilcoxon signed-rank test. Boldface type indicates statistical significance. Effect sizes were calculated using Hedges *g* (N < 50) for preoperative to 1-year comparisons in this group, as group sizes fell below 50. ACL-R, anterior cruciate ligament reconstruction; ADL, Activities of Daily Living; ES, effect size; KOOS, Knee injury and Osteoarthritis Outcome Score; KOOS_4_, mean of the Symptoms, Pain, Sports and Recreation, and Quality of Life subscales; QoL, Quality of Life; Sports/Rec, Sports and Recreation.

### Clinical and Surgical Failure

Ipsilateral ACL revision within 1 year was recorded in 1.1% (n = 2) of the ACL-R + LET group and 1.0% (n = 7) of the ACL-R group (*P* > .99). Within 2 years, ipsilateral ACL revision rates were 2.3% (n = 4) in the ACL-R + LET group and 2.4% (n = 17) in the ACL-R group (*P* > .99).

Rates of clinical failure (KOOS QoL <44) within 1 year were 30.8% (n = 20) in the ACL-R + LET group and 30.2% (n = 91) in the ACL-R group (*P* > .99). At 2 years, rates improved to 25.5% (n = 12) and 25.6% (n = 60), respectively (*P* > .99).

## Discussion

The principal finding of this matched-cohort analysis from the SKLR was that the addition of LET to primary ACL-R did not demonstrate superior short-term PROs. Both groups achieved clinically meaningful within-group KOOS improvements from preoperative baseline, exceeding the MIC thresholds for Sports/Rec and KOOS_4_, corroborating that primary ACL-R provides clinically meaningful benefit to the patient regardless of LET augmentation. The matched cohort was adequately powered to detect a 10-point between-group difference in KOOS_4_; accordingly, the null finding for the primary KOOS_4_ outcome was therefore robust and was unlikely to represent a type 2 error. Overall, there were low rates of revision ACL-R (2.3% in ACL-R + LET, 2.4% in ACL-R); however, the study was not powered to detect differences in surgical or clinical failure.

The broader LET literature has predominantly evaluated knee laxity and graft failure outcomes rather than PROs. The STABILITY trial enrolled exclusively young patients (14-25 years) with at least 2 of 3 defined high-risk features (competitive pivoting sports participation, grade ≥2 pivot shift, and generalized ligamentous laxity) and demonstrated a clinically meaningful reduction in composite ACL-R clinical failure.^9^ The present registry cohort applied no high-risk inclusion criteria, and the mean age of approximately 25 years in both groups, while consistent with a younger population, does not distinguish high-risk from low-risk phenotypes. A clinically important residual imbalance in injury setting persisted after matching despite the caliper procedure (14% in the ACL-R + LET group vs 6.9% in the ACL-R group for other setting), as injury setting was not a specified matching variable. Patients with nonpivoting or injuries in other settings who nonetheless received LET may represent off-indication recipients whose inclusion could have attenuated any group-level PRO benefit. Furthermore, preoperative pivot-shift grade, Beighton hypermobility score, tibial slope, and competitive sports level, all key determinants of high-risk designation, are not captured in the SKLR, precluding risk stratification. The differential recovery trajectory also merits consideration: the LET group did not achieve a significant improvement in KOOS Symptoms at 1 year, with significant further gains in Symptoms, QoL, and KOOS_4_ from 1 to 2 years, a pattern not observed in the ACL-R group. This may reflect early soft tissue morbidity from the additional anterolateral incision, postoperative pain, or a longer period of healing required for the lateral tenodesis construct, rather than an inferior ultimate outcome. Importantly, somewhat paradoxically, the LET group did have higher KOOS Symptoms values (ie, were less symptomatic) preoperatively. While LET is typically performed for specific objective criteria (as opposed to symptomatology), it is possible the LET cohort who underwent surgery later (although not significantly) after injury may have benefitted from a greater duration of higher-quality physiotherapy due to being clustered at later dates in the study period. These observations collectively suggest that the null PRO finding is not inconsistent with the STABILITY trial data, but instead likely reflects the heterogeneous, predominantly non–high-risk composition of the registry cohort.

A central implication of these findings pertains to patient selection for LET. A recent systematic review identified high-risk sports activity, concurrent medial meniscal repair or excision, and grade 2 or 3 pivot shift as the most frequently cited indications across 29 published studies.^32^ None of these high-risk variables are captured in the SKLR, making it impossible to determine what proportion of LET recipients in this cohort were treated in accordance with these evidence-based indications. Accordingly, the findings of the current study suggest that the PRO benefit of LET is likely contingent on rigorous patient selection in accordance with established evidence-based indications and reinforce the need for national registries to prospectively capture high-risk clinical variables to enable risk-stratified analyses that can more precisely define the subpopulations most likely to benefit from this adjunct procedure. Importantly, the current study is subject to selection/indication bias: LET was presumed to be used in patients with unmeasured high-risk features (pivot-shift grade, generalized ligamentous laxity, return-to–pivoting sports status) that are not captured by the SKLR and could not be included in the matching algorithm. As such, the equivalent PRO and revision findings observed here should not be generalized to argue against LET in the high-risk subpopulations in whom it is currently indicated.

The present study offers important strengths. The use of a large prospective nationwide registry with standardized data collection ensures generalizability to the real-world ACL-R population across a diverse range of surgeons and institutions. While Sweden's universal health care system and relatively homogeneous population differentiate it from other countries, patient characteristics, sports participation, and revision rates are generally comparable when evaluating registry data.^22^ Additionally, factors affecting PROs within the SKLR (eg, male sex, nonsmoking status, absence of concomitant injuries) are similar to those reported in other studies.^12,28,29^ The decreased use of LET in the SKLR likely represents its sparing use in the highest-risk patients, who would likely benefit the most from the procedure. The 1:4 matching procedure balanced all specified confounders across all matching variables. Within-group KOOS improvements exceeded MIC thresholds for Sports/Rec and KOOS_4_ in both groups, confirming the clinical meaningfulness of the postoperative gains observed, independent of between-group differences. While the current analysis does not report return-to-sports rates, KOOS Sports/Rec accounts for up to 70% of variation in sports function, and is a validated instrument for assessing self-reported sports function after ACL-R.^4,5,27^ The a priori power analysis demonstrates that the null finding for the primary KOOS_4_ outcome was adequately powered and cannot be dismissed as a consequence of insufficient sample size. Finally, the inclusion of nonhamstring graft types appropriately reflects sports medicine practices; however, future prospective studies will be critical in characterizing the utility and outcomes of LET procedures in diverse cohorts undergoing nonhamstring ACL-R.

Several study limitations must be acknowledged. First, despite 1:4 caliper matching on key clinical confounders, the observational design precludes randomization, and residual confounding by indication, most notably by unmeasured high-risk variables, cannot be eliminated. Second, the LET group was relatively small (n = 174), a unique finding in a registry of >20,000 patients, likely reflecting a mixture of selective uptake of LET within the SKLR during the study period and insufficient follow-up duration due to increasing adaptation in the last 1 to 2 years. As such, the current sample size was not powered to detect differences in revision ACL-R rates. Third, the SKLR registry only had data for patients undergoing LET with the modified Lemaire technique, so the generalizability to other techniques is poorly characterized. Fourth, the study period spans 2 decades (2005-2025), during which ACL-R and LET techniques and fixation methods, patient selection criteria, and rehabilitation protocols underwent substantial evolution. Importantly, inclusion of the LET technique as a variable in the SKLR began in 2016, and earlier LET cases were performed in a markedly different evidence landscape than cases after publication of the STABILITY trial in 2020.^9^ A sensitivity analysis restricted to the post-2020 era, when current consensus criteria were more firmly established, was not feasible given the small LET group size (174 total), but such an analysis would be valuable in future work as registry LET numbers accumulate.

Next, attrition was a limitation of the current study, with 2-year PRO response rates of 27.0% in the ACL-R + LET arm and 33.6% in the ACL-R arm, consistent with 2-year response rates typical of national knee ligament registries.^8,23^ We did not perform a formal comparison of baseline characteristics between those who completed the 2-year questionnaire and those who did not, and we therefore cannot directly quantify the potential for nonrandom attrition to bias the 2-year PRO estimates. Several features of the study design partially mitigate this concern but do not eliminate it. The cohort was assembled by 1:4 caliper matching, constraining imbalance on these measured prognostic factors. Prior analyses of the SKLR have not identified systematic nonresponse bias on these variables. Furthermore, the revision ACL-R outcome is captured at the population level through the Swedish personal identity number and is therefore available for all 870 matched patients regardless of questionnaire return. Finally, objective stability measures (pivot shift, arthrometer laxity) were not captured by the SKLR, precluding assessment of the primary mechanistic outcome of LET.

## Conclusion

The addition of LET to primary ACL-R did not demonstrate superior PROs at 1 or 2 years compared with ACL-R alone. Both groups achieved clinically meaningful improvements in patient-reported knee function from preoperative baseline, supporting the clinical effectiveness of primary ACL-R regardless of LET augmentation.
